# Seasonal Variations in Habitat Use are Associated With Food Availability Changes in Assamese Macaques (*Macaca assamensis*) Inhabiting Limestone Forest

**DOI:** 10.1002/ece3.70629

**Published:** 2024-12-04

**Authors:** Fengxiang Mo, Guanghua Liu, Ailong Wang, Shengyuan Liu, Shixin Nong, Kechu Zhang, Zhonghao Huang

**Affiliations:** ^1^ Key Laboratory of Ecology of Rare and Endangered Species and Environmental Protection (Guangxi Normal University), Ministry of Education Guilin China; ^2^ Guangxi Key Laboratory of Rare and Endangered Animal Ecology Guangxi Normal University Guilin China; ^3^ Key Laboratory of Genetic Evolution & Animal Models Kunming Institute of Zoology, Chinese Academy of Sciences Kunming Yunnan China; ^4^ Administration Center of Guangxi Nonggang National Nature Reserve Longzhou China; ^5^ Key Laboratory of Mountain Biodiversity Conservation, Education Department of Guangxi Zhuang Autonomous Region Yulin Normal University Yulin China

**Keywords:** Assamese macaques, food availability, habitats use, limestone forest, *Macaca assamensis*, seasonality

## Abstract

Data on habitat use of wild animals facilitate conservation management and further our understanding of their environmental adaptations. We collected data on the habitat utilization of 16 groups of Assamese macaques (*Macaca assamensis*) in the limestone forests in Guangxi, China, to explore the pattern and seasonality of habitat use of these macaques. Our results showed that cliffs were the most frequently used hill parts by the macaques, followed by flat zones, hillsides, and hilltops. The cliffs were the most frequently used during resting and moving, whereas the hillsides and flat zones were used as the main feeding sites. Patterns of habitat use seasonally varied. Specifically, the utilization frequency of the cliff was lower in the rainy season than in the dry season. Besides, feeding occurred more frequently on hillsides and flat zones. The dietary composition of the Assamese macaques affected the use of hill parts, indicating that the consumption of young leaves was positively correlated with the use of cliffs and was passively related to the use of hillsides. Moreover, ecological factors had impacts on habitat use. The use of hilltops and cliffs increased whereas the utilization of hillsides and flat zones decreased with the day length and temperature dropping. The availability of flower and fruit were also key ecological factors affecting habitat utilization. We conclude that the dietary composition, day length, flower availability, and fruit availability are determinants of habitat utilization for Assamese macaques, highlighting the importance of ecological factors in shaping their behavioral adaptation to the unique limestone forests.

## Graphical abstract

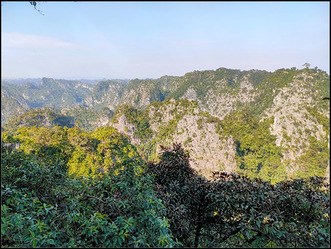

## 1 Introduction

Behavioral mechanism reflects the adaptation and evolution of animals to the environment (Overdorff 1996; Cowlishaw 1997; Hill 2006; Zhou et al. 2013; Terada et al. 2015), and the habitat utilization patterns are commonly linked to the flexibility of their adaptation to habitats (Marsh and Chapman 2013). The habitat use of animals is affected by various effects, including distribution and availability of food resources (Hanya et al. 2006; Li et al. 2020a), predation risk (Li et al. 2021), group size (O'Brien and Kinnaird 1997; Zhao 1999; Riley 2008), intergroup competition (Kurihara and Hanya 2018), habitat structure (Albani et al. 2020), and climate change (Hill et al. 2004; Li et al. 2021). Among these factors, the distribution and availability of food resources are key factors affecting animal habitat utilization (Riley 2008; Santhosh et al. 2015). Due to variations in food availability in both temporal and spatial contexts (Huang et al. 2010; Li et al. 2016), the food resources available to wild animals commonly differ in various microhabitats, resulting in fluctuations in habitat use (Liu et al. 2022). For example, animals tend to frequently forage in areas with higher abundance of food resources (O'Brien and Kinnaird 1997), as reported in the cases of the Assamese macaques (*Macaca assamensis*) (Li, Zhou, and Huang 2017; Li et al. 2021) and the François' langurs (*Trachypithecus francoisi*) (Chen et al. 2019).

Predation risk is a significant factor affecting habitat selection by animals (Coleman and Hill 2014). Commonly, primates tend to use safe habitats that have low predation pressures (Cowlishaw 1997). For example, François' langurs choose the cliffs as sleeping sites to avoid predators (Zhou, Cai, and Huang 2010), and Angolan colobus monkeys (*Colobus angolensis ruwenzorii*) choose to rest in larger trees and higher tree canopies to reduce the risk of predation (Adams and Teichroeb 2020). However, Barbary macaques (*Macaca sylvanus*) are primarily concerned with foraging benefits, and foraging for crops despite the frequent appearances of local farmers (Namous and Znari 2018). Thus, the habitat utilization of primates is also thought to be a result of the balance between food availability and predation risk (Cowlishaw 1997; Enstam and Isbell 2004; Li, Zhou, and Huang 2017; Trapanese, Meunier, and Masi 2019), as access to food resources and predators avoidance are important components of most animal survival strategies (Cowlishaw 1997).

Behavioral thermoregulation strategy in response to climate changes significantly shapes the habitat utilization of primates, which is essential to maintain constant body temperatures (Hanya, Kiyono, and Hayaishi 2007; McFarland et al. 2020). For this purpose, primates have evolved specific adaptations to the seasonal changes in climates (Reed and Fleagle 1995; Mendiratta et al. 2009). These behavioral strategies include reducing heat production or accelerating heat dissipation in hot environments (McFarland et al. 2020), as well as increasing heat production in cold conditions or reducing heat loss through behavioral thermal regulation (McFarland and Majolo 2013). Due to the differences in the microclimate across various microhabitats, the temperature changes are expected to force animals to selectively occupy their habitats (Li et al. 2021). For example, both Assamese macaques and white‐headed langurs (*Trachypithecus leucocephalus*) use the hilltops to bask in the sun during the cold months, which is considered as an effective strategy for reducing the cost of behavioral thermoregulation (Huang et al. 2000; Li et al. 2021).

Assamese macaques are predominantly distributed in the South and Southeast Asia (Boonratana et al. 2020). Assamese macaques living in the limestone forests of Guangxi are highly folivores and heavily depend on the young leaves of a karst endemic plant, *Bonia amplexicaulis*, throughout the year (Wada et al. 2010; Huang et al. 2015). Previous studies show that Assamese macaques adjust their dietary composition according to seasonal changes in food availability, preferring fruits during the fruit‐rich season (rainy season) and increasing leaves consumption during the fruit‐lean season (dry season) (Huang et al. 2016). Compared to the populations living in tropical forests, Assamese macaques inhabiting limestone forests have shorter daily ranging distances and smaller home ranges, which are linked to their low energy expenditures (Li et al. 2020a). There is still limited research on the habitat utilization of Assamese macaques in the limestone forests. Previous studies on Assamese macaques in Nonggang show that the seasonal change of habitat utilization is impacted by food distribution and temperature (Li et al. 2021). However, information on the habitat use pattern of these macaques living in other limestone forests have been not available yet. In this study, we collected data on habitat use of the Assamese macaques inhabiting Longrui forest. We described the patterns of habitat use of macaques and then explored their seasonal variations in Longrui area. Finally, we examined the effects of ecological factors on habitat use. We tested the following predictions.

### 1.1 Prediction 1

The vigilance of primates would reduce during stationary activities (Cords 1995). During resting and grooming, they are more likely to choose areas with relatively low predation risk (Cowlishaw 1997). In the limestone forests, the hilltops and the cliffs are covered by sparse vegetation which are considered as relatively safe places (Zhou et al. 2013). Therefore, we predicted that the Assamese macaques would use the cliffs for resting more frequently than other parts of the limestone forests.

### 1.2 Prediction 2

There are spatial differences in the distribution of vegetation in karst areas, with marked variations in the vegetation among different hill parts (Liang et al. 1985; Tan 2014), which could result in differences in habitat utilization by animals due to the heterogeneous distribution of vegetation. Normally, most of the vegetation and food resources are distributed in the flat zones of the limestone hills (Liu et al. 2022). Therefore, we predicted that the Assamese macaques would use flat zones as feeding sites more frequently than other parts of the limestone hills.

### 1.3 Prediction 3

In the limestone forests of Guangxi, China, it is hot and humid in the rainy season but cold and dry in the dry season (Li, Zhou, and Huang 2017; Liu et al. 2024). The adaptation of animals to seasonal fluctuations in climatic factors commonly leads to changes in the thermoregulation strategies of animals, consequently affecting their habitat utilization (McFarland et al. 2020; Eppley et al. 2022; Hilário et al. 2022). For example, Assamese macaques in Nonggang choose to avoid high temperatures in the rainy season by resting on the cool hillside, and they bask on the bare rock surface near the sleeping sites in the dry season (Li et al. 2021). Thus, we predicted that the Assamese macaques would less frequently use hilltops and cliffs, and more frequently use hillsides and flat zones during the rainy season than those in the dry season.

### 1.4 Prediction 4

Previous study has shown that the proportion of young leaves in the diet of the Assamese macaques in limestone forests is higher than other geographical macaque groups, whereas the proportion of fruits is lower than other geographical macaque groups; moreover, the dietary composition of Assamese macaques is affected by temperature (Huang et al. 2016). The food resources of Assamese macaques are significantly affected by seasonal changes, with the availability of fruits and young leaves varying seasonally (Huang et al. 2015). Therefore, we predicted that the availability of young leaves and fruits would affect the habitat utilization of the Assamese macaques.

## 2 Methods

### 2.1 Study Site

This study was carried out in the Guangxi Nonggang National Nature Reserve (106°42′28″–107°04′54″ E, 22°13′56″–22°33′09″ N) from September 2021 to September 2022. The reserve is located in the southwest of Guangxi, China, spanning Longzhou and Ningming counties, including Nonggang (5424.7 hm^2^), Longrui (1008.0 hm^2^), and Longshan (3644.8 hm^2^) (Tan 2014). This study was conducted in the center of Longrui Area.

Located in the south of the Tropic of Cancer (Li et al. 2021), the reserve has a tropical monsoon climate with dry and rainy seasons throughout the year. During the study period, we collected climatic data such as temperature, humidity, rainfall, and day length of this study site. The results showed that the annual mean daily length was 12.15 h, and the daily length varied from 10.82 h in January to 13.46 h in July. The total annual rainfall was 924.30 mm, and the mean monthly rainfall was 77.00 ± 49.90 mm. The maximum rainfall in June 2022 was 157.80 mm, and the minimum in December 2021 was 5.40 mm. According to the amount of mean monthly rainfall, we divided the whole year into the rainy season (May to October, the mean monthly rainfall was 115.30 ± 34.00 mm) and the dry season (November to April, the mean monthly rainfall was 38.80 ± 30.00 mm) (Liu et al. 2024).

**FIGURE 1:**
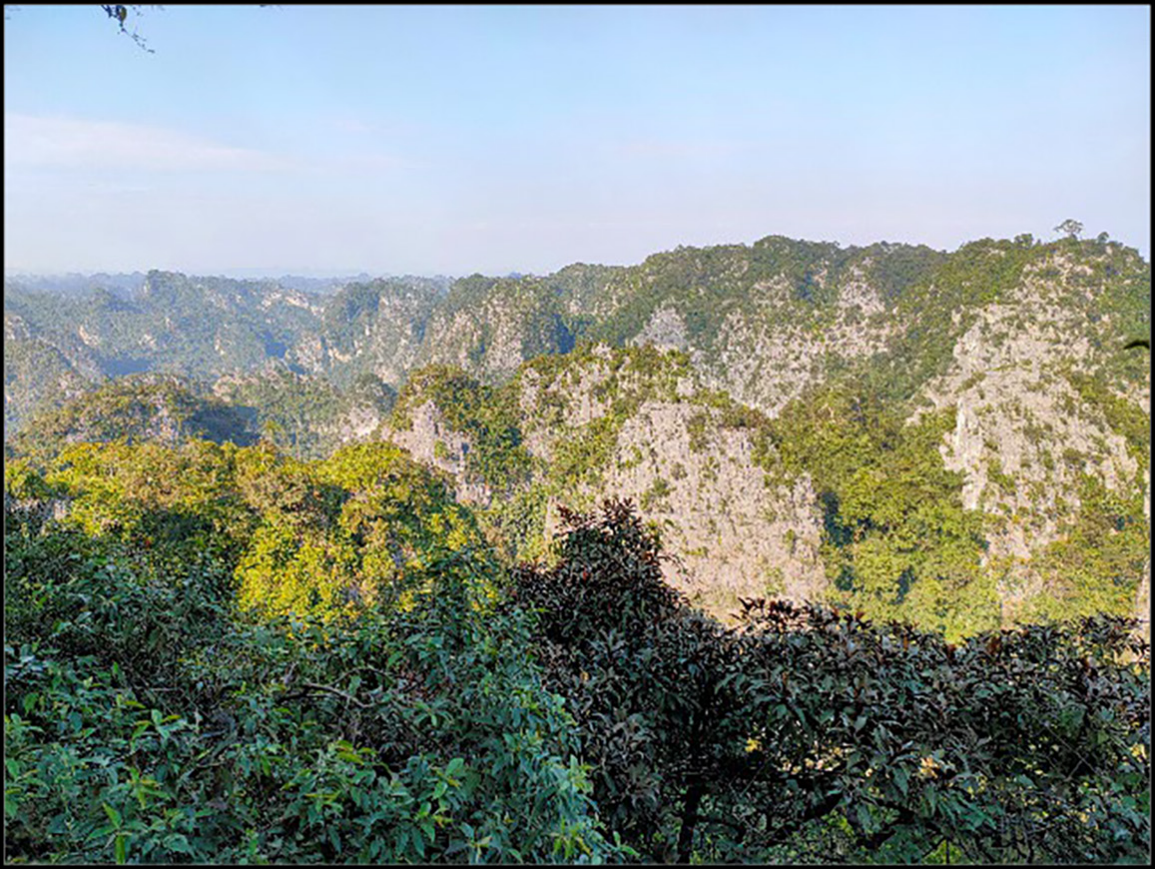
Habitats of the Assamese macaques in the current study.

The reserve features typical karst landforms, mainly consisting of peak depressions and peak valleys, with an elevation ranging from 300 m to 700 m (Tan 2014). The vegetation in this area is mainly limestone mountain monsoon rainforest, mostly covered with tropical tall trees and vines. The plants have typical xerophytic characteristics. Due to the conditions of moisture and soil, these plants have significant characteristics of drought tolerance. Considering the topography and vegetation distribution, we roughly divide the hills into four parts: hilltops, cliffs, hillsides, and flat zones (Figure 1).

### 2.2 Vegetation Survey

To assess the woody plant species composition, we investigated the vegetation in the main study area with the quadrat method. According to the distribution of the mountain vegetation, we set up 20 quadrats (20 × 20 m) according to the proportion of different hill parts in the habitat, including three on the hilltops, eight on the hillsides, and nine on the flat zones. We could not set plots in the cliffs due to their inaccessibility. During the survey, we recorded the species and number of woody plants in the quadrats, measured the diameter at breast height (DBH > 3 cm) of each woody plant, and recorded the width of each canopy and relevant crown height. When plant species could not be identified on‐site, specimens were collected, recorded, and brought back to the laboratory for further identification.

### 2.3 Behavioral Data Collection

We totally recorded behavioral data from 16 groups of Assamese macaques (Table S1). We observed these macaques within a distance of 5–200 m. This method has little effect on the macaques' habitat use (Workman and Schmitt 2012). We randomly selected observation macaque group. Behavioral sampling was performed from the initial sighting of focal group until nightfall when the macaques returned into their sleeping sites or we lost contact with them for more than 30 min. We collected data using instantaneous scan sampling. Scans began every 15 min and lasted for 5 min. During scanning, we scanned from the left to the right to avoid potential bias toward given individuals. We divided the behaviors of the observed macaques into feeding, resting, and moving. Feeding was recorded when foraging, picking, ingestion, and chewing the food by the majority of individuals in the group, including the short‐distance movements during foraging. Resting referred to the unchanged position of the macaque individual. Moving included bridging, quadrupedal walking, quadrupedal running, climbing, and leaping (Hunt et al. 1996; Chen et al. 2020; Qiu et al. 2024). From October 2021 to September 2022, we conducted 148 days of follow‐up observation. We collected 2028 scans, which resulted in a total of 8172 individual records and 2685 foraging records.

### 2.4 Data Analysis

We processed the obtained woody plants data to calculate DBH area of woody plants: DBH area = π × (DBH of the woody plant)^2^/4.

We calculated the relative coverage (RC), relative density (RD), and relative frequency (RF) to assess the dominance of various woody plants as follows (Zheng et al. 2021): Dominance = RC+ RD + RF

$$\text{Relative coverage}=\left(\frac{\mathrm{DBH}\;\text{area of given woody plant}}{\mathrm{DBH}\;\text{area of}\;\mathrm{all}\;\text{woody plants}}\right)\times 100\%$$

$$\text{Relative density}=\left(\frac{\text{number of trees of given woody plant species}}{\text{total number of trees of}\;\mathrm{all}\;\text{woody plant species}}\right)\times 100\%$$

$$\text{Relative frequency}=\left(\frac{\text{frequency of given woody plant}}{\text{frequency of}\;\mathrm{all}\;\text{woody plants}}\right)\times 100\%$$

The biomass of woody plants was represented by the crown volume of woody plants: Crown volume = (basal area × crown height)/3; Basal area = π × (crown width)^2^/4.

We selected the Shannon‐Wiener index to represent the diversity of woody plant species for different hill parts, and the formula used was: $H^{\prime }$ = − $\sum \left(P_{i}\times \ln\left(P_{i}\right)\right)$; where $P_{i}$ = $N_{i}$/*N*, represented the proportion of plant *i* in the woody plant recorded, and *N* represented the total number of all plants in the quadrat. After calculating the Shannon‐Wiener index, we set different hill parts into groups and test the normal distribution using a Kolmogorov–Smirnov test. The data did not follow a normal distribution. Kruskal‐Waillis test was used to test the difference between groups.

We expressed the dietary composition using proportion of feeding time devoted to various food parts and species (Huang et al. 2016; Liu et al. 2024). Specifically, we first calculated dietary composition by dividing the individual numbers that were recorded as feeding in each scan by the number of the total feeding members and then averaged these proportions to determine the hourly data. The monthly dietary composition was obtained by averaging the hourly dietary compositions. We calculated the seasonal and annual habitat use based on the averages of relevant months' dietary compositions. The Food Availability Index (FAI) of the food parts was calculated respectively, the calculation formula was as follows: FAI = $\sum_{i=1}^{n}D_{i}B_{i}P_{i}$. $D_{i}$ represented the density of tree species, $B_{i}$ represented the base coverage of tree species, and $P_{i}$ represented the phenological scores of food parts (Huang et al. 2015). To collect phenological scores, we selected 20 food species mainly eaten by Assamese macaques for phenological monitoring. We monitored 10 individuals of each plant and observed the growth of food parts (young leaves, flowers, fruits, and other parts), and assigned values from 0 to 4 to indicate the proportion of food parts in the tree crown (0: 0%, 1: 0.1%–25%, 2: 25.1%–50%, 3: 50.1%–75%, 4: 75.1%–100%).

We used the Kolmogorov–Smirnov test to test the normality of the variables. The results showed that some variables were not normally distributed. Thus, we used the Mann–Whitney U test to detect differences between two groups and the Kruskal‐Wallis H test to analyze differences among multiple groups. Considering inter‐season variable differences and the influence of sample size on the results, we generated generalized linear mixed models (GLMMs) to compare seasonal differences in habitat utilization with the random effects (Huang et al. 2017; Chen et al. 2020; Qiu et al. 2024). We used habitat utilization data as the response variable, the number of macaques observed per month as the random factor, and the season as the fixed factor. We tested the differences between the models with and without fixed factors using ANOVA to determine the role of fixed factors in the model. When the *p* value was < 0.05, “season” was considered as a significant factor shaping the model's goodness of fit.

We generated generalized linear models (GLMs) to perform model averaging to examine the effects of dietary composition and ecological factors on habitat use (Li et al. 2020a, 2020b; Zhang et al. 2023). To improve linearity and normality, we converted the data of habitat utilization using a logit transformation (Warton and Hui 2011; Li et al. 2020b). To assess the relative importance (*W*_*ip*_) of each variable in the model, we employed multi‐model inference based on Akaike Information Criterion (AICc) with a small sample size correction. The *W*_*ip*_ was obtained by summing the relative importance of each model that contains the predictor variables (Liu et al. 2014; Li et al. 2015; Xu et al. 2017). Due to the day length was highly correlated with average temperature and the young leaves FAI (day length and average temperature: *r* = 0.965, *n* = 12, *p* < 0.001; day length and young leaves FAI: *r* = 0.860, *n* = 12, *p* < 0.001), we constructed two models (models I and II) to analyze the impact of ecological factors on habitat utilization (Li et al. 2020b, 2021). Model I included day length but excluded average temperature and young leaf FAI, while model II included average temperature and young leaf FAI but excluded day length.

The significance level of all tests was set at 0.05. Data processing and analysis were performed on the Microsoft Excel 2016, SPSS 22.0, and R 4.3.3. The GLMM models were grouped on the *lmer* function from the lme4 package (Douglas Bates, Bolker, and Walker 2015; Kamil 2023). The multi‐model inferences were based on GLM model using the *dredge* and *model.avg.* functions of the MuMIn package (Li et al. 2020b).

## 3 Results

### 3.1 Dominance of Woody Plants at the Study Site

A total of 1166 woody plant species were recorded in all 20 quadrats. A total of 988 woody plants belonging to 37 families and 79 species were identified. At the family level, Euphorbiaceae had the most species (8 species with 134 individuals), followed by Moraceae (7 species with 227 individuals) and Malvaceae (6 species with 132 individuals). These three families accounted for 26.58% and 42.28% of the total species and plant counts, respectively. *Streblus tonkinensis* had the highest number of individuals (184 individuals), followed by *Cleidion brevipetiolatum* (82 individuals) and *Sterculia monosperma* (72 individuals). In this study area, *Dracontomelon duperreanum* exhibited the highest dominance, *Streblus tonkinensis* had the highest number of individuals, and *Celtis sinensis* had the largest biomass (Table 1).

**TABLE 1:** Dominances of predominated woody plants in the study site.

Species	Family	Number of individual	Relative coverage (%)	Relative density (%)	Relative frequency (%)	Dominance (%)	Biomass/m^3^
*Dracontomelon duperreanum*	Anacardiaceae	39	25.0	3.3	1.8	30.1	3080.5
*Streblus tonkinensis*	Moraceae	184	3.3	15.7	2.9	22.0	2477.5
*Sterculia monosperma*	Malvaceae	72	3.2	6.2	4.2	13.6	1320.7
*Celtis sinensis*	Ulmaceae	16	9.1	1.4	2.1	12.5	4230.9
*Vitex kwangsiensis*	Lamiaceae	42	3.5	3.6	3.2	10.2	1830.2
*Cleidion brevipetiolatum*	Euphorbiaceae	82	0.9	7.0	1.8	9.7	547.9
*Microcos paniculata*	Tiliaceae	22	4.1	1.9	2.6	8.6	2386.2
*Bischofia javanica*	Euphorbiaceae	29	2.9	2.5	2.9	8.3	1389.3
*Garuga forrestii*	Burseraceae	9	3.5	0.8	1.6	5.8	1320.4
*Arenga westerhoutii*	Arecoideae	8	3.4	0.7	1.1	5.1	246.4

**FIGURE 2:**
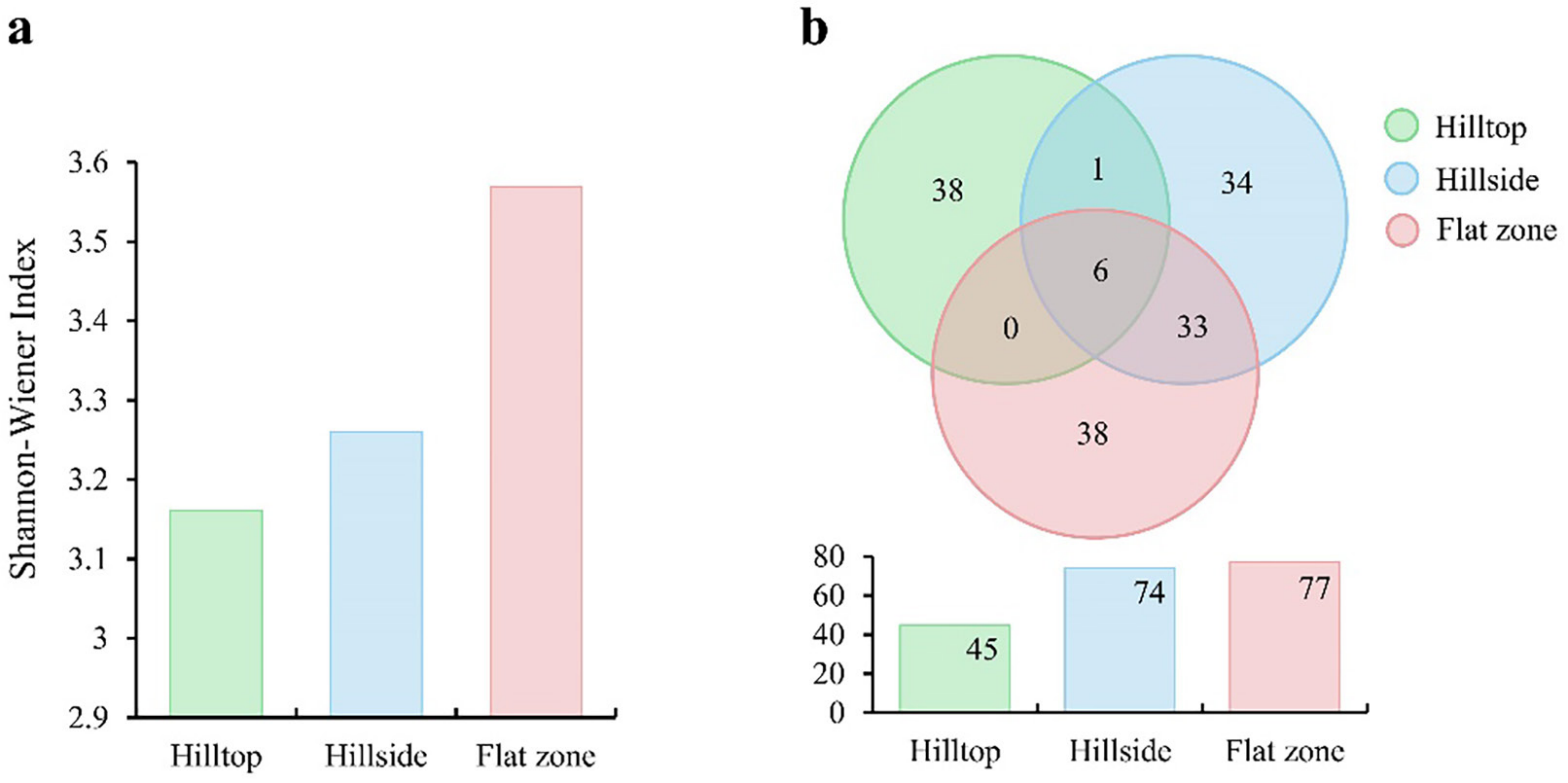
Shannon–Wiener index of woody plants in different hill parts of habitat (a); the number of shared and specific woody plants in different hill parts (b).

According to the vegetation survey results, the Shannon‐Wiener index had no significant difference (*H* = 1.394, df = 2, *p* = 0.498). The flat zone had the largest value (3.57), followed by the hillside (3.26) and the hilltop (3.16) (Figure 2a). *Bonia amplexicaulis* was mainly distributed on cliffs with a slope of 80°–90° but limitedly grew in other hill parts, which was the favorite food for Assamese macaques. It was difficult to carry out vegetation surveys on cliffs, thus no vegetation distribution information was collected. In the hilltops, *Sinosideroxylon pedunculatum* had the largest number of trees (26 individuals), followed by *Pistacia weinmanniifolia* (19 individuals), and *Memecylon scutellatum* (16 individuals). *Sinosideroxylon pedunculatum* had the highest dominance, the largest number of trees, and the largest biomass. *Pistacia weinmanniifolia*, *Psydrax dicocca, Sinosideroxylon pedunculatum, and Boniodendron minus* did not occur in other hill parts (Figure 2b, Table S2). In the hillsides, *Streblus tonkinensis* had the most trees (133 individuals), followed by *Cleidion brevipetiolatum* (27 individuals) and *Sterculia monosperma* (20 individuals). *Streblus tonkinensis* had the highest dominance, the largest number of trees, and the largest biomass on the hillside (Figure 2b, Table S3). In the flat zones, the largest number of trees was *Cleidion brevipetiolatum* (55 individuals), followed by *Sterculia monosperma* (51 individuals) and *Streblus tonkinensis* (51 individuals). *Dracontomelon duperreanum* had the highest dominance and the largest biomass (Figure 2b, Table S4).

### 3.2 Habitat Utilization and Seasonal Differences

**FIGURE 3:**
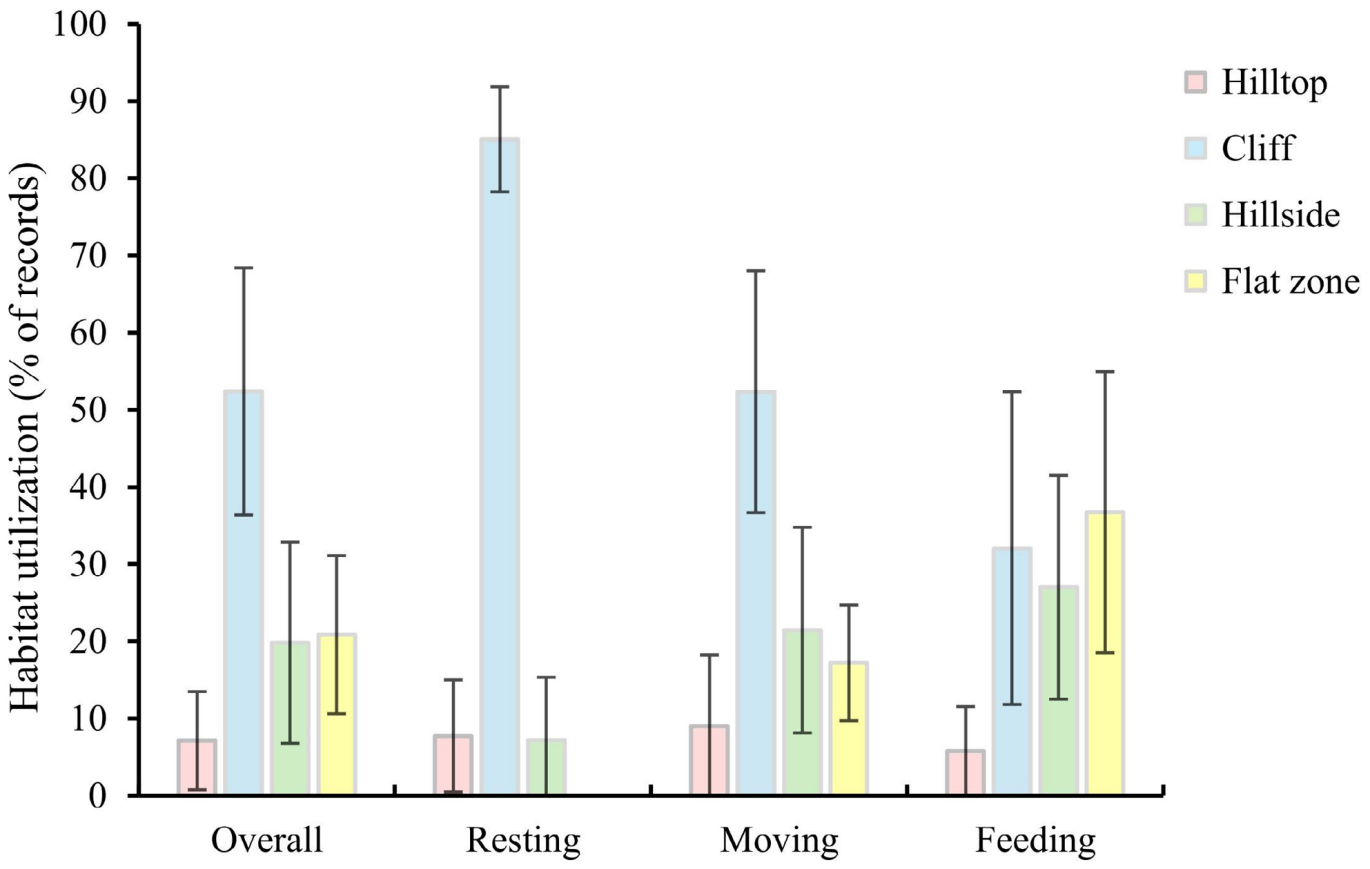
Utilization of the hill parts used by Assamese macaques in the whole year.

There were significant differences in habitat utilization of hill parts during the whole year by Assamese macaques (*χ* ^2^ = 30.135, df = 3, *p* < 0.001). The cliffs (52.38% ± 16.02%) were the most frequently used parts of the limestone hills, followed by the flat zones (20.86% ± 10.26%), hillsides (19.82% ± 13.03%), and hilltops (7.12% ± 6.37%) (Figure 3).

There were differences in the use of hill parts in specific activity by Assamese macaques (Resting: *χ* ^2^ = 36.445, df = 3, *p* < 0.001; Moving: *χ* ^2^ = 27.972, df = 3, *p* < 0.001; Feeding: *χ* ^2^ = 19.812, df = 3, *p* < 0.001) (Figure 3). Specifically, cliffs were the most frequently used zone during resting (85.07% ± 6.81%), followed by hilltops (7.74% ± 7.25%) and hillsides (7.19% ± 8.15%). When macaques moving, cliffs were the top used zone (52.35% ± 15.68%), followed by hillsides (21.46% ± 13.32%), flat zones (17.20% ± 7.50%), and hilltops (9.00% ± 9.25%). When feeding, these macaques predominantly used flat zones (36.72% ± 18.21%), followed by cliffs (32.07% ± 20.65%), hillsides (27.01% ± 14.50%), and hilltops (5.78% ± 5.75%).

**FIGURE 4:**
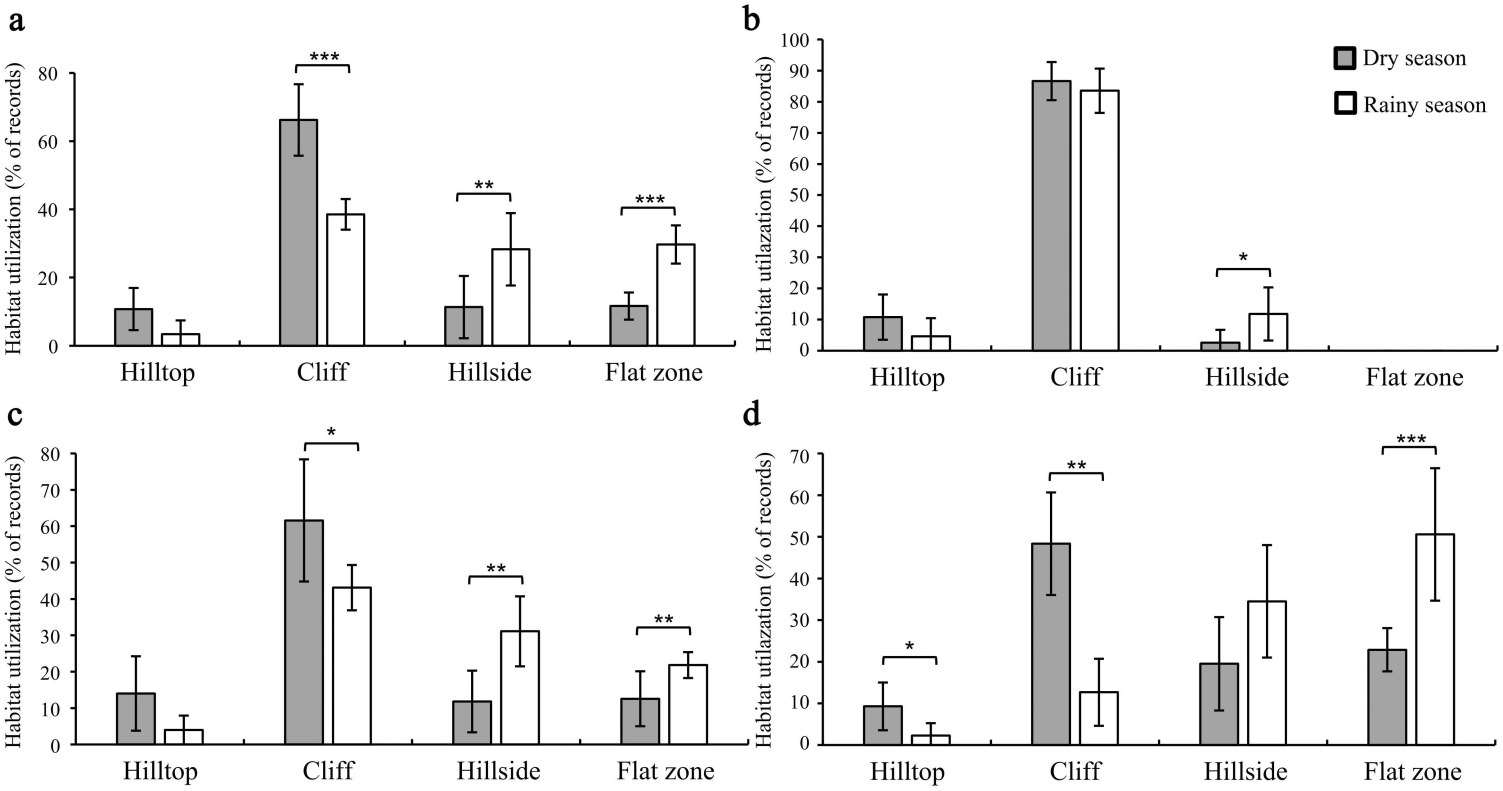
Seasonal differences in the utilization of the hill parts used by Assamese macaques during specific activity (a: Overall, b: Resting, c: Moving, d: Feeding; *p < 0.05, **p < 0.01, ***p < 0.001).

Seasonally, the utilization frequency of the cliffs during the observation period was significantly higher in the dry season than in the rainy season, whereas the utilization frequency of hillsides and flat zones was significantly higher in the rainy season than in the dry season. However, there was no significant seasonal difference in the utilization frequency of hilltops (Figure 4, Table S5). In addition, there were significant seasonal differences in the utilization of the hill parts during specific activities. During resting, the use frequency of hillsides was higher in the rainy season than in the dry season; however, the use frequency of hilltops and cliffs was not significantly different (Figure 4, Table S5). During moving, the use frequency of cliffs was higher in the dry season than in the rainy season, whereas the utilization of hillsides and flat zones was higher in the rainy season than in the dry season. However, there was no significant seasonal difference in the utilization of hilltops (Figure 4, Table S5). During feeding, the use frequency of hillsides and flat zones was higher in the dry season than in the rainy season, reversing the pattern observed for hilltops and cliffs. There were significant seasonal differences in the utilization of flat zones.

### 3.3 Dietary Composition and Habitat Utilization

Habitat utilization of the Assamese macaques was influenced by their dietary composition (Table 2, Table S6). The utilization of cliffs was positively correlated with the consumption of young leaves (*β* = 0.670, *W*_*ip*_ = 0.98). The utilization of hillsides was negatively correlated with the consumption of young leaves (*β* = −0.549, *W*_*ip*_ = 0.82) and positively correlated with the consumption of flowers (*β* = 0.150, *W*_*ip*_ = 0.90). Moreover, the use frequency of the flat zones decreased when the proportion of young leaves in their diet increased (*β* = −0.708, *W*_*ip*_ = 0.99). Dietary composition had no significant effect on the utilization of hilltops.

**TABLE 2:** Effect of dietary composition on habitat utilization of the Assamese macaques, based on GLM model averaging.

Explanatory variables	*β*	*SE*	*Z*	*p*	95% CI		*W*_*ip*_
Hilltop							
Intercept	−2.139	0.747	0.863	0.004	−3.603	−0.674	0.18
Young leaves	1.444	0.868	1.664	0.096	−0.256	3.146	0.42
Mature leaves	−0.273	0.361	0.775	0.450	−0.982	0.435	0.13
Flowers	−0.247	0.225	1.094	0.274	−0.689	0.195	0.19
Fruits	−0.252	0.302	0.834	0.404	−0.845	0.340	0.14
Stems	−0.276	0.234	1.179	0.238	−0.735	0.182	0.20
Cliff							
Intercept	−0.106	0.087	1.218	0.223	−0.227	0.064	0.00
Young leaves	0.670	0.119	5.634	< 0.001	0.437	0.904	0.98
Mature leaves	0.001	0.046	0.042	0.967	−0.088	0.092	0.06
Flowers	−0.039	0.027	1.460	0.114	−0.092	0.013	0.30
Fruits	−0.005	0.045	0.123	0.902	−0.094	0.083	0.06
Stems	−0.007	0.034	0.209	0.835	−0.075	0.060	0.07
Hillside							
Intercept	−0.248	0.205	1.211	0.225	−0.651	0.153	0.01
Young leaves	−0.549	0.210	2.618	0.008	−0.961	−0.138	0.82
Mature leaves	−0.043	0.100	0.434	0.664	−0.240	0.153	0.07
Flowers	0.150	0.052	2.883	0.003	0.048	0.253	0.90
Fruits	0.103	0.099	1.047	0.295	−0.090	0.297	0.12
Stems	0.021	0.087	0.241	0.809	−0.149	0.191	0.06
Flat zone							
Intercept	−0.518	0.060	8.552	< 0.001	−0.637	−0.400	0.00
Young leaves	−0.708	0.090	7.846	< 0.001	−0.885	−0.531	0.99
Mature leaves	0.016	0.033	0.487	0.626	−0.049	0.082	0.08
Flowers	0.020	0.025	0.837	0.403	−0.028	0.069	0.13
Fruits	−0.011	0.032	0.341	0.733	−0.074	0.052	0.07
Stems	−0.025	0.025	1.011	0.312	−0.074	0.023	0.16

Abbreviations: *β*, regression coefficient; 95% CI, 95% confidence interval for *β*; FAI, food availability index; *W*_*ip*_, the relative importance of the variable.

### 3.4 Ecological Factors and Habitat Utilization

The utilization of hill parts by the Assamese macaques was affected by ecological factors (Tables 3 and 4, Tables S7 and S8). The result of model I (Table 3) showed that day length was the key factor affecting the utilization of hilltops, cliffs, and hillsides. The utilization of hilltops (*β* = −22.381, *W*_*ip*_ = 0.44) and cliffs (*β* = −7.516, *W*_*ip*_ = 0.93) was negatively correlated with day length, whereas the utilization of hillsides (*β* = 11.266, *W*_*ip*_ = 0.96) was positively correlated with day length. The utilization of the flat zones was negatively correlated with flower FAI (*β* = −0.185, *W*_*ip*_ = 0.41), and the utilization of the flat zones was positively correlated with fruit FAI (*β* = 0.757, *W*_*ip*_ = 0.60) and day length (*β* = 6.762, *W*_*ip*_ = 0.39).

**TABLE 3:** Effect of ecological factors on habitat utilization of the Assamese macaques, based on GLM model averaging (Model I).

Explanatory variables	*β*	*SE*	*Z*	*p*	95% CI		*W*_*ip*_
Hilltop							
Intercept	26.521	40.791	0.650	0.515	−53.428	106.471	0.04
Mature leaf FAI	9.918	5.108	1.942	0.052	−0.092	19.930	0.41
Flower FAI	−0.344	0.483	0.712	0.476	−1.290	0.602	0.10
Fruit FAI	−1.281	2.140	0.598	0.549	−5.476	2.914	0.10
Average humidity	−2.008	2.375	0.846	0.397	−6.663	2.646	0.13
Rainfall	−0.678	1.026	0.661	0.508	−2.690	1.333	0.09
Day length	−22.381	11.332	1.975	0.048	−44.592	−0.170	0.44
Cliff							
Intercept	19.793	8.305	2.383	0.017	3.515	36.071	0.00
Mature leaf FAI	−0.471	1.493	0.316	0.751	−3.398	2.454	0.07
Flower FAI	0.101	0.114	0.882	0.377	−0.123	0.325	0.13
Fruit FAI	−0.439	0.342	1.281	0.200	−1.111	0.232	0.27
Average humidity	0.246	0.361	0.682	0.495	−0.462	0.955	0.12
Rainfall	−0.106	0.214	0.495	0.620	−0.527	0.314	0.07
Day length	−7.516	2.085	3.605	< 0.001	−11.603	−3.429	0.93
Hillside							
Intercept	−32.137	9.088	3.536	< 0.001	−49.950	−14.324	0.00
Mature leaf FAI	−1.895	1.212	1.563	0.117	−4.271	0.480	0.34
Flower FAI	0.034	0.077	0.448	0.654	−0.116	0.186	0.05
Fruit FAI	−0.446	0.440	1.014	0.310	−1.310	0.417	0.10
Average humidity	−0.558	0.508	1.098	0.272	−1.555	0.438	0.12
Rainfall	−0.258	0.154	1.672	0.094	−0.562	0.044	0.34
Day length	11.266	2.885	3.904	< 0.001	5.610	16.923	0.96
Flat zone							
Intercept	−8.473	11.645	0.728	0.466	−31.297	14.351	0.00
Mature leaf FAI	1.555	1.668	0.932	0.351	−1.714	4.826	0.10
Flower FAI	−0.185	0.090	2.047	0.040	−0.363	−0.007	0.41
Fruit FAI	0.757	0.343	2.205	0.027	0.084	7.430	0.60
Average humidity	0.200	0.768	0.262	0.793	−1.304	1.706	0.10
Rainfall	0.352	0.219	1.605	0.108	−0.077	0.782	0.25
Day length	6.762	3.308	2.044	0.041	0.277	13.247	0.39

Abbreviations: *β*, regression coefficient; 95% CI, 95% confidence interval for *β*; FAI, food availability index; *W*_*ip*_, the relative importance of the variable.

**TABLE 4:** Effect of ecological factors on habitat utilization of the Assamese macaques, based on GLM model averaging (Model II).

Explanatory variables	*β*	*SE*	*Z*	*p*	95% CI		*W*_*ip*_
Hilltop							
Intercept	−2.143	5.241	0.409	0.682	−12.417	8.129	0.04
Young leaf FAI	−3.747	0.792	1.342	0.179	−9.221	1.726	0.30
Mature leaf FAI	10.098	2.524	1.828	0.067	−0.729	20.925	0.41
Flower FAI	−0.171	0.458	0.374	0.708	−1.070	0.727	0.06
Fruit FAI	−1.308	2.181	0.600	0.548	−5.584	2.967	0.10
Mean temperature	−4.287	3.588	1.195	0.232	−11.319	2.744	0.19
Average humidity	−2.225	2.258	0.985	0.324	−6.652	2.201	0.16
Rainfall	−0.806	0.915	0.881	0.378	−2.600	0.987	0.10
Cliff							
Intercept	0.201	1.268	0.159	0.873	−2.284	2.688	0.00
Young leaf FAI	−1.081	0.782	1.383	0.100	−2.614	0.451	0.26
Mature leaf FAI	0.299	2.846	0.105	0.916	−5.278	5.877	0.08
Flower FAI	0.180	0.082	2.179	0.029	0.018	0.343	0.47
Fruit FAI	−0.788	0.316	2.496	0.012	−1.408	−0.169	0.56
Mean temperature	−1.310	0.935	1.402	0.161	−3.143	0.521	0.19
Average humidity	0.056	0.705	0.080	0.936	−1.326	1.439	0.07
Rainfall	−0.302	0.206	1.466	0.142	−0.706	0.101	0.21
Hillside							
Intercept	−1.249	0.851	1.467	0.142	−2.919	0.420	0.00
Young leaf FAI	2.097	0.493	4.247	< 0.001	1.129	3.065	0.86
Mature leaf FAI	−2.481	2.037	1.218	0.223	−6.474	1.512	0.16
Flower FAI	−0.082	0.065	1.268	0.205	−0.211	0.045	0.20
Fruit FAI	−0.337	0.277	1.215	0.224	−0.882	0.206	0.17
Mean temperature	1.318	0.919	1.434	0.152	−0.483	3.120	0.20
Average humidity	0.294	0.354	0.832	0.405	−0.339	0.988	0.11
Rainfall	0.131	0.157	0.839	0.402	−0.176	0.439	0.09
Flat zone							
Intercept	−0.881	1.370	0.643	0.520	−3.568	1.805	0.01
Young leaf FAI	1.103	1.479	0.746	0.455	−1.795	4.002	0.09
Mature leaf FAI	2.446	3.220	0.766	0.443	−3.884	8.778	0.09
Flower FAI	−0.192	0.083	2.303	0.021	−0.356	−0.028	0.50
Fruit FAI	0.803	0.325	2.466	0.013	0.164	1.442	0.68
Mean temperature	1.150	1.009	1.140	0.254	−0.827	3.129	0.15
Average humidity	0.268	0.806	0.333	0.739	−1.312	1.849	0.10
Rainfall	0.339	0.211	1.891	0.058	−0.014	0.814	0.37

Abbreviations: *β*, regression coefficient; 95% CI, 95% confidence interval for *β*; FAI, food availability index; *W*_*ip*_, the relative importance of the variable.

The result of model II (Table 4) showed that FAI of flower and fruit were the key factors influencing cliffs use. Specifically, the utilization of cliffs was positively correlated with flower FAI (*β* = 0.180, *W*_*ip*_ = 0.47), and negatively correlated with fruit FAI (*β* = −0.788, *W*_*ip*_ = 0.56). Moreover, young leaves was the key factor affecting the utilization of the hillsides and showed a positive correlation with utilization frequency (*β* = 2.097, *W*_*ip*_ = 0.86). The utilization of the flat zones was negatively correlated with flower FAI (*β* = −0.192, *W*_*ip*_ = 0.50) and was positively correlated with fruit FAI (*β* = 0.803, *W*_*ip*_ = 0.68). However, there was no significant influence of ecological factors on the utilization of hilltops.

## 4 Discussion

In this study, there were significant differences in the utilization frequency of different hill parts in the habitat by Assamese macaques, and the performance of specific behaviors in various hill parts significantly differed. These macaques spent most of their resting and moving time on the cliffs and devoted most of their feeding time to the flat zones (Figure 3), which completely supports our Predictions 1, and 2. The results of the vegetation survey showed that the preferred food of Assamese macaques was more frequently distributed in the hillsides and flat zones (Table 1, Tables S2–, S4). Thus, Assamese macaques spend most of their foraging time on the hillsides and flat zones which likely contribute to the foraging efficiency. Especially in the rainy season when fruit resources are relatively abundant, Assamese macaques reduce the intake of leaves and spend more time searching for fruits on the hillsides and flat zones (Li, Zhou, and Huang 2017; Li et al. 2018, 2021), consequently making hillsides and flat zones the top used as foraging areas (Huang et al. 2000; Chen et al. 2019; Li et al. 2021). As found in other limestone primates, food availability affects the utilization of hill parts by François' langurs in Nonggang, Guangxi (Zhou et al. 2013; Chen et al. 2019). The fruits and seeds eaten by François' langurs are mostly distributed in the flat zones, and rainfall is positively correlated with the quantity of fruits, young leaves, and other food parts (Huang et al. 2008; Ting, Hartley, and Burns 2008). Therefore, the langurs increase the utilization of the flat zones with increasing consumptions of fruits and seeds (Chen et al. 2019). However, the results of Li et al. (2021) are not similar to our findings, showing that Assamese macaques rarely go down to the flat zones; instead, they forage more frequently on the hillsides, and then directly rest in the foraging area there. Due to human activities occurred in the flat zones occupied by farmers, it is difficult for Assamese macaques in Nonggang to forage on the flat zones. Staying in high places such as hillsides facilitates hiding and detecting predators more quickly when predators approach (Li et al. 2021). The environment in Longrui is relatively primitive, with almost no human interference. Affected by the distribution of soil and water, food resources in different hill parts are relatively dispersed and patchy (Liang et al. 1985; Li et al. 2018), whereas trees and vines on the hillsides and flat zones are abundant (Liang et al. 1985; Su, Zhao, and Huang 1988), providing more foraging options for Assamese macaques. Moreover, we found that after leaving the sleeping sites in the morning, these macaques spend most time feeding in the hillsides and flat zones. After foraging they returned to the middle of the cliffs or stayed in the hillsides for resting. However, no macaques were recorded to rest in the flat zones.

In addition, based on existing research findings, primates tend to choose a safe place for resting, grooming, and other activities due to the decreased vigilance during these activities (Cords 1995; Cowlishaw 1997). For safety reasons, the steep cliffs and hilltops markedly reduce the predation risk (Huang et al. 2003). Several plants are exclusively distributed on the hilltops and cliffs which shelter these karst‐dwelling primates to avoid predators (Zhou et al. 2013). These could be linked to the high frequency of the cliffs used for the limestone‐dwelling Assamese macaques during resting. Other primates in the limestone forests are similar to the Longrui Assamese macaques that also prefer cliffs to decrease predation risk during stationary activities (Huang et al. 2000; Chen et al. 2019; Li et al. 2021).

There were seasonal variations in the utilization of hill parts by Assamese macaques. The utilization of cliffs for resting and feeding in the dry season was higher than that in the rainy season. They most frequently rested and fed on the hillsides and flat zones in the rainy season. The result strongly supports Prediction 3. Seasonal fluctuations in climatic factors commonly lead to changes in the thermoregulation strategies of animals, consequently affecting their habitat utilization (Hanya, Kiyono, and Hayaishi 2007; McFarland et al. 2020). In the dry season without sunshine, the wind on the hilltops and cliffs is strong, and the surface temperature of the bare rock is low (Larson, Matthes, and Kelly 2000). Assamese macaques hide in the crevices and caves of the cliffs to avoid the wind and maintain body temperature. When the weather is warmer in the dry season, Assamese macaques use the cliffs more frequently, which could be linked to sunbathing in low temperatures to reduce the cost of thermoregulation (Huang et al. 2003). When temperatures drop in the dry season, primates bask in sunlight to raise body surface temperature and reduce energy expenditure (Li et al. 2021). For example, François' langurs sunbathe to absorb and retain heat by huddling together on the cliffs in winter (dry season) (Li, Huang, and Huang 2020). The same results have been found in the study of Li et al. (2021), showing that temperature affects the habitat use by Assamese macaques. When the sun is shining in winter, the temperature in the southern direction of the mountain is generally higher than in other directions, making macaques more susceptible to direct sunlight and to getting more heat energy (Larson, Matthes, and Kelly 2000). These macaques use different slopes of the cliffs to change their thermal regulation strategies and reduce energy loss in the dry season by basking on bare rock to obtain heat energy and maintain constant body temperature (Kelly et al. 2016). In the rainy season, food is mainly distributed on the hillsides and flat zones, resting on these areas after foraging can reduce energy loss. It can also avoid direct sunlight and reduce body surface temperature (Liang et al. 1985; Li et al. 2021). In this study, Assamese macaques resting on the hillsides can reduce sun exposure and avoid high temperatures in the rainy season (Adams and Teichroeb 2020; Li, Huang, and Huang 2020). Similarly, the wild Vervet monkeys (*Chlorocebus pygerythrus*) spend more time in the shade reducing their body heats (McFarland et al. 2020). Therefore, seasonal variations in the food resource and climate could be the factor influencing the habitat utilization of Assamese macaques.

According to the results, the use of hillsides by Assamese macaques was positively correlated with young leaves. Fruit was positively correlated with flat zones use and negatively correlated with cliffs use (Tables 2, 3, 4, Tables S6–, S8). These results support Prediction 4. The studies indicate that the Assamese macaques living in the limestone forests show marked characteristics of leaf feeding (Huang et al. 2015, 2016; Liu et al. 2024). In our study, the proportions of young leaves and fruits in macaques' diets were 57.91% ± 21.47% and 32.95% ± 23.49%, respectively (Liu et al. 2024). There were significant differences in the proportion of young leaves and fruits between different seasons, which showed that the proportion of fruits in the rainy season was higher than that in the dry season, and the proportion of young leaves in the dry season was higher than that in the rainy season (Liu et al. 2024). Due to the obvious seasonal changes and the uneven distribution of food resources among hill parts lead to the changes in dietary composition of Assamese macaques (Liang et al. 1985; Su, Zhao, and Huang 1988; Huang et al. 2015, 2016). In the rainy season, fruits, young leaves, and flowers are abundant and mainly distributed in the hillsides and flat zones (Liang et al. 1985; Su, Zhao, and Huang 1988; Huang et al. 2015, 2016). Assamese macaques prefer fruits and devote a large amount of foraging time to harvest fruits in the rainy season (Huang et al. 2015). Thus, the utilization of hillsides and flat zones increase. In the dry season when fruits and young leaves in shortage, Assamese macaques collect mature leaves and young leaves of the *Bonia amplexicaulis* as main and stable foods, which are mainly distributed in the cliffs (Huang et al. 2015). Compared to foraging on the hillsides or flat zones, foraging on the cliffs in the dry season could decrease energy expenditure (Huang et al. 2015). Additionally, it is relatively safer to forage in the cliffs (Li et al. 2021). Therefore, the availability of young leaves and fruits affects the utilization of hill parts by Assamese macaques.

## 5 Conclusions

This study aims to further understand the adaptive strategies of the Assamese macaques in the limestone forests and enrich the results of the behavioral ecology of the Assamese macaques. Our study provides information on the habitat utilization patterns of Assamese macaques in Guangxi. The results suggest that the distribution of food resources, and ecological factors might be the driving factors influencing the habitat utilization of Assamese macaques. Seasonal variations in food resources and differences in spatial distribution affect their choice of foraging areas, and the predation risk and temperature influence their behavioral choices, which in turn lead to seasonal differences in the habitat use of Assamese macaques. We conclude that the habitat use patterns of Assamese macaques in the limestone forests could be linked to the food availability, predator avoidance, and behavioral thermoregulation of these macaques. This highlights the importance of the ecological factors on their behavioral responses to the changing environment and underscores the need to understand the interactions between these ecological factors and their adaptations to the limestone forests.

## Author Contributions

**Fengxiang Mo:** data curation (equal), writing – original draft (equal). **Guanghua Liu:** data curation (equal), investigation (equal), supervision (equal). **Ailong Wang:** investigation (equal). **Shengyuan Liu:** investigation (equal). **Shixin Nong:** investigation (equal). **Kechu Zhang:** funding acquisition (equal), investigation (equal), project administration (equal). **Zhonghao Huang:** conceptualization (equal), funding acquisition (equal), project administration (equal), writing – review and editing (equal).

## Conflicts of Interest

The authors declare no conflicts of interest.

## Supporting information


**Table S1** Group sizes of the Assamese macaques in Longrui, Guangxi.

**Table S2** Dominances of predominated woody plants on the hilltop.

**Table S3** Dominances of predominated woody plants on the hillside.

**Table S4** Dominances of predominated woody plants on the flat zone.

**Table S5** Seasonal variations in the hill parts used by Assamese macaques during various activities.

**Table S6** The candidate models of the effect of dietary composition on habitat utilization in Assamese macaques based on GLM model (ΔAIC ≤ 2).

**Table S7** The candidate models of the effect of ecological factors on habitat utilization in Assamese macaques based on GLM model I (ΔAIC ≤ 2).

**Table S8** The candidate models of the effect of ecological factors on habitat utilization in Assamese macaques based on GLM model II (ΔAIC ≤ 2).

## Data Availability

All data are available in the figshare repository at https://doi.org/10.6084/m9.figshare.25471159.v1.
