# Supplementary material for: Seasonal Variations in Habitat Use are Associated With Food Availability Changes in Assamese Macaques (Macaca assamensis) Inhabiting Limestone Forest
Source: Ecol Evol. 2024 Dec 4;14(12):e70629. doi: 10.1002/ece3.70629 (PMC11617327; doi:10.1002/ece3.70629)
Supplement: Supplementary file 1 — Table S1 Group sizes of the Assamese macaques in Longrui, Guangxi. [file ECE3-14-e70629-s003.docx]

Table S1 Group sizes of the Assamese macaques in Longrui, Guangxi

| **Group ID** | **Group size** | **Observation days** | **Number of scans** |
| --- | --- | --- | --- |
| Group 1 | 22 | 51 | 683 |
| Group 2 | 15 | 1 | 11 |
| Group 3 | 14 | 14 | 120 |
| Group 4 | > 23 | 1 | 1 |
| Group 5 | > 25 | 1 | 12 |
| Group 6 | > 20 | 1 | 13 |
| Group 7 | 20 | 8 | 109 |
| Group 8 | 9 | 1 | 1 |
| Group 9 | > 20 | 20 | 135 |
| Group 10 | 7 | 6 | 21 |
| Group 11 | > 15 | 45 | 356 |
| Group 12 | 21 | 5 | 28 |
| Group 13 | 16 | 18 | 175 |
| Group 14 | 18 | 3 | 13 |
| Group 15 | > 23 | 10 | 113 |
| Group 16 | > 25 | 24 | 219 |
