# Supplementary material for: Seasonal Variations in Habitat Use are Associated With Food Availability Changes in Assamese Macaques (Macaca assamensis) Inhabiting Limestone Forest
Source: Ecol Evol. 2024 Dec 4;14(12):e70629. doi: 10.1002/ece3.70629 (PMC11617327; doi:10.1002/ece3.70629)
Supplement: Supplementary file 2 — Table S2 Dominances of predominated woody plants on the hilltop. [file ECE3-14-e70629-s001.docx]

Table S2 Dominances of predominated woody plants in the hilltop

| **Species** | **Family** | **Number** | **Relative coverage (%)** | **Relative density (%)** | **Relative frequency (%)** | **Dominance (%)** | **Biomass /m^3^** |
| --- | --- | --- | --- | --- | --- | --- | --- |
| *Sinosideroxylon* *pedunculatum* | Sapotaceae | 26 | 24.4 | 16.3 | 5.4 | 46.0 | 478.7 |
| *Pistacia weinmannifolia* | Anacardiaceae | 19 | 16.5 | 11.9 | 5.4 | 33.8 | 485.4 |
| *Boniodendron minus* | Sapindaceae | 7 | 15.0 | 4.4 | 3.6 | 22.9 | 110.7 |
| *Memecylo scutellatum* | [*Melastomataceae*](http://www.iplant.cn/info/Melastomataceae) | 16 | 6.5 | 10.0 | 5.4 | 21.8 | 69.0 |
| *Cleistanthus sumatranus* | Phyllanthaceae | 7 | 2.8 | 4.4 | 1.8 | 9.0 | 39.1 |
| *Dracaena cochinchinensis* | Asparagaceae | 5 | 1.9 | 3.1 | 3.6 | 8.6 | 2.9 |
| *Diospyros siderophylla* | [Ebenaceae](http://www.iplant.cn/info/Ebenaceae?t=z) | 4 | 2.0 | 2.5 | 3.6 | 8.1 | 36.1 |
| *Psydrax dicocca* | Rubiaceae | 4 | 2.4 | 2.5 | 1.8 | 6.7 | 61.9 |
