# Supplementary material for: Seasonal Variations in Habitat Use are Associated With Food Availability Changes in Assamese Macaques (Macaca assamensis) Inhabiting Limestone Forest
Source: Ecol Evol. 2024 Dec 4;14(12):e70629. doi: 10.1002/ece3.70629 (PMC11617327; doi:10.1002/ece3.70629)
Supplement: Supplementary file 3 — Table S3 Dominances of predominated woody plants on the hillside. [file ECE3-14-e70629-s006.docx]

Table S3 Dominances of predominated woody plants in the hillside

| **Species** | **Family** | **Number** | **Relative coverage (%)** | **Relative density (%)** | **Relative frequency (%)** | **Dominance (%)** | **Biomass /m^3^** |
| --- | --- | --- | --- | --- | --- | --- | --- |
| *Streblus tonkinensis* | [Moraceae](http://www.iplant.cn/info/Moraceae?t=z) | 133 | 5.2 | 27.4 | 4.0 | 36.6 | 1879.0 |
| *Dracontomelon duperreanum* | [Anacardiaceae](http://www.iplant.cn/info/Anacardiaceae?t=z) | 2 | 24.2 | 0.4 | 1.3 | 25.9 | 205.1 |
| *Sterculia monosperma* | Malvaceae | 20 | 2.8 | 4.1 | 4.6 | 11.5 | 504.1 |
| *Microcos paniculata* | [Tiliaceae](http://www.iplant.cn/info/Tiliaceae?t=z) | 6 | 4.9 | 1.2 | 3.3 | 9.4 | 716.2 |
| *Vitex kwangsiensis* | Lamiaceae | 15 | 3.0 | 3.1 | 3.3 | 9.4 | 790.5 |
| *Celtis sinensis* | [Ulmaceae](http://www.iplant.cn/info/Ulmaceae?t=z) | 5 | 6.2 | 1.0 | 2.0 | 9.3 | 1788.9 |
| *Cleidion brevipetiolatum* | [Euphorbiaceae](http://www.iplant.cn/info/Euphorbiaceae?t=z) | 27 | 0.6 | 5.6 | 2.6 | 8.9 | 216.4 |
| *Arenga westerhoutii* | Arecaceae | 6 | 5.8 | 1.2 | 1.3 | 8.4 | 220.3 |
| *Orophea polycarpa* | [Annonaceae](http://www.iplant.cn/info/Annonaceae?t=z) | 19 | 0.3 | 3.9 | 3.3 | 7.5 | 127.4 |
