# Supplementary material for: Seasonal Variations in Habitat Use are Associated With Food Availability Changes in Assamese Macaques (Macaca assamensis) Inhabiting Limestone Forest
Source: Ecol Evol. 2024 Dec 4;14(12):e70629. doi: 10.1002/ece3.70629 (PMC11617327; doi:10.1002/ece3.70629)
Supplement: Supplementary file 4 — Table S4 Dominances of predominated woody plants on the flat zone. [file ECE3-14-e70629-s002.docx]

Table S4 Dominances of predominated woody plants in the flat zone

| **Species** | **Family** | **Number** | **Relative coverage (%)** | **Relative density (%)** | **Relative frequency (%)** | **Dominance (%)** | **Biomass /m^3^** |
| --- | --- | --- | --- | --- | --- | --- | --- |
| *Dracontomelon duperreanum* | [Anacardiaceae](http://www.iplant.cn/info/Anacardiaceae?t=z) | 37 | 29.0 | 7.1 | 3.0 | 39.2 | 2875.4 |
| *Sterculia monosperma* | Malvaceae | 51 | 4.0 | 9.8 | 4.8 | 18.6 | 816.0 |
| *Celtis sinensis* | [Ulmaceae](http://www.iplant.cn/info/Ulmaceae?t=z) | 11 | 13.2 | 2.1 | 2.4 | 17.7 | 2442.0 |
| *Streblus tonkinensis* | [Moraceae](http://www.iplant.cn/info/Moraceae?t=z) | 51 | 1.8 | 9.8 | 3.0 | 14.6 | 598.5 |
| *Vitex kwangsiensis* | Lamiaceae | 27 | 4.5 | 5.2 | 4.2 | 13.9 | 1039.7 |
| *Bischofia javanica* | Euphorbiaceae | 22 | 5.2 | 4.2 | 4.2 | 13.7 | 1184.0 |
| *Cleidion brevipetiolatum* | [Euphorbiaceae](http://www.iplant.cn/info/Euphorbiaceae?t=z) | 55 | 1.2 | 10.6 | 1.8 | 13.6 | 331.5 |
| *Garuga forrestii* | [Burseraceae](http://www.iplant.cn/info/Burseraceae?t=z) | 7 | 6.5 | 1.3 | 2.4 | 10.2 | 1234.4 |
| *Microcos paniculata* | [Tiliaceae](http://www.iplant.cn/info/Tiliaceae?t=z) | 16 | 3.8 | 3.1 | 3.0 | 9.8 | 1670.0 |
| *Ficus hispida* | Moraceae | 26 | 0.7 | 5.0 | 3.0 | 8.7 | 444.9 |
