# Supplementary material for: Seasonal Variations in Habitat Use are Associated With Food Availability Changes in Assamese Macaques (Macaca assamensis) Inhabiting Limestone Forest
Source: Ecol Evol. 2024 Dec 4;14(12):e70629. doi: 10.1002/ece3.70629 (PMC11617327; doi:10.1002/ece3.70629)
Supplement: Supplementary file 5 — Table S5 Seasonal variations in the hill parts used by Assamese macaques during various activities. [file ECE3-14-e70629-s007.docx]

Table S5 Seasonal variations in the hill parts used by Assamese macaques during various activities

| **Activity behavior** | **Statistic** | **Hill parts** | | | |
| --- | --- | --- | --- | --- | --- |
|  |  | **Hilltop** | **Cliff** | **Hillside** | **Flat zone** |
| Overall | χ^2^ (*df* = 1) | 3.716 | 15.182 | 6.806 | 17.277 |
|  | *P* | 0.053 | < 0.001^***^ | 0.009^**^ | < 0.001^***^ |
| Resting | χ^2^ (*df* = 1) | 3.568 | 0.030 | 6.601 | - |
|  | *P* | 0.058 | 0.950 | 0.010^*^ | - |
| Moving | χ^2^ (*df* = 1) | 1.156 | 4.494 | 9.286 | 6.955 |
|  | *P* | 0.282 | 0.026^*^ | 0.002^**^ | 0.008^**^ |
| Feeding | χ^2^ (*df* = 1) | 4.621 | 7.181 | 3.045 | 11.547 |
|  | *P* | 0.031^*^ | 0.007^**^ | 0.080 | < 0.001^***^ |

“^*^” indicate significant differences: ^*^ P < 0.05; ^**^ P < 0.01; ^***^ P < 0.001. “-” indicate the data was not collected.
