# Supplementary material for: Seasonal Variations in Habitat Use are Associated With Food Availability Changes in Assamese Macaques (Macaca assamensis) Inhabiting Limestone Forest
Source: Ecol Evol. 2024 Dec 4;14(12):e70629. doi: 10.1002/ece3.70629 (PMC11617327; doi:10.1002/ece3.70629)
Supplement: Supplementary file 6 — Table S6 The candidate models of the effect of dietary composition on habitat utilization in Assamese macaques based on GLM model (ΔAIC ≤ 2). [file ECE3-14-e70629-s005.docx]

Table S6 The candidate models of the effect of dietary composition on habitat utilization in Assamese macaques based on GLM model (ΔAIC ≤ 2)

| Variable | Hilltop | | | | Cliff | | Hillside | Flat zone |
| --- | --- | --- | --- | --- | --- | --- | --- | --- |
|  | Model 1 | Model 2 | Model 3 | Model 4 | Model 1 | Model 2 | Model 1 | Model 1 |
| Intercept |  | ● |  |  |  |  |  |  |
| Young leaves | ● |  |  |  | ● | ● | ● | ● |
| Mature leaves |  |  |  |  |  |  |  |  |
| Flower |  |  |  | ● |  | ● | ● |  |
| Fruit |  |  |  |  |  |  |  |  |
| Stems |  |  | ● |  |  |  |  |  |
| AICc | 41.80 | 42.21 | 43.27 | 43.63 | -5.57 | -4.41 | 9.77 | -12.91 |
| ΔAIC | 0.00 | 0.41 | 1.47 | 1.82 | 0.00 | 1.34 | 0.00 | 0.00 |
| W*_i_* | 0.22 | 0.18 | 0.10 | 0.09 | 0.52 | 0.27 | 0.66 | 0.61 |

●: variable included in the model; AICc: Akake’s information criterion corrected for small sample sizes; ΔAIC: difference between specific model and most high-ranked one; W*_i_*: Akaike weights, the probability that a model is best given the particulai set of models considered.
