# Supplementary material for: Seasonal Variations in Habitat Use are Associated With Food Availability Changes in Assamese Macaques (Macaca assamensis) Inhabiting Limestone Forest
Source: Ecol Evol. 2024 Dec 4;14(12):e70629. doi: 10.1002/ece3.70629 (PMC11617327; doi:10.1002/ece3.70629)
Supplement: Supplementary file 7 — Table S7 The candidate models of the effect of ecological factors on habitat utilization in Assamese macaques based on GLM model I (ΔAIC ≤ 2). [file ECE3-14-e70629-s008.docx]

Table S7 The candidate models of the effect of ecological factors on habitat utilization in Assamese macaques based on GLM model Ⅰ (ΔAIC ≤ 2)

| Variable | Hilltop | | Cliff | Hillside | | | | Flat zone | |
| --- | --- | --- | --- | --- | --- | --- | --- | --- | --- |
|  | Model 1 | Model 2 | Model 1 | Model 1 | Model 2 | Model 3 | Model 4 | Model 1 | Model 2 |
| Mature leaves FAI | ● |  |  |  |  | ● | ● |  |  |
| Flower FAI |  |  |  |  |  |  |  | ● |  |
| Fruit FAI |  |  |  |  |  |  |  | ● |  |
| Average humidity |  |  |  |  |  |  |  |  |  |
| Rainfall |  |  |  |  | ● |  | ● |  |  |
| Day length |  | ● | ● | ● | ● | ● | ● |  | ● |
| AICc | 38.55 | 38.63 | -2.30 | -1.53 | -1.16 | -0.78 | 0.36 | 2.38 | 3.53 |
| ΔAIC | 0.00 | 0.07 | 0.00 | 0.00 | 0.37 | 0.76 | 1.89 | 0.00 | 1.14 |
| W*_i_* | 0.51 | 0.49 | 0.93 | 0.34 | 0.29 | 0.24 | 0.13 | 0.64 | 0.36 |

●: variable included in the model; AICc: Akake’s information criterion corrected for small sample sizes; ΔAIC: difference between specific model and most high-ranked one; W*_i_*: Akaike weights, the probability that a model is best given the particulai set of models considered.
