# Supplementary material for: Seasonal Variations in Habitat Use are Associated With Food Availability Changes in Assamese Macaques (Macaca assamensis) Inhabiting Limestone Forest
Source: Ecol Evol. 2024 Dec 4;14(12):e70629. doi: 10.1002/ece3.70629 (PMC11617327; doi:10.1002/ece3.70629)
Supplement: Supplementary file 8 — Table S8 The candidate models of the effect of ecological factors on habitat utilization in Assamese macaques based on GLM model II (ΔAIC ≤ 2). [file ECE3-14-e70629-s004.docx]

Table S8 The candidate models of the effect of ecological factors on habitat utilization in Assamese macaques based on GLM model Ⅱ (ΔAIC ≤ 2)

| Variable | Hilltop | | Cliff | Hillside | | | Flat zone |
| --- | --- | --- | --- | --- | --- | --- | --- |
|  | Model 1 | Model 2 | Model 1 | Model 1 | Model 2 | Model 3 | Model 1 |
| Young leaves FAI |  | ● |  | ● | ● | ● |  |
| Mature leaves FAI | ● |  |  |  |  |  |  |
| Flower FAI |  |  | ● |  | ● |  | ● |
| Fruit FAI |  |  | ● |  |  | ● | ● |
| Average humidity |  |  |  |  |  |  |  |
| Rainfall |  |  |  |  |  |  |  |
| Average temperature |  |  |  |  |  |  |  |
| AICc | 38.63 | 39.63 | 2.18 | -0.66 | 0.84 | 1.19 | 2.38 |
| ΔAIC | 0.00 | 1.01 | 0.00 | 0.00 | 1.49 | 0.84 | 0.00 |
| W*_i_* | 0.62 | 0.38 | 0.32 | 0.53 | 0.25 | 0.21 | 0.35 |

●: variable included in the model; AICc: Akake’s information criterion corrected for small sample sizes; ΔAIC: difference between specific model and most high-ranked one; W*_i_*: Akaike weights, the probability that a model is best given the particulai set of models considered.
